# Local Medical Societies

**Published:** 1889-10

**Authors:** 


### LOCAL MEDICAL SOCIETIES.

With the advent of cooler weather, the time has arrived when medical societies all over the land are buckling on the armor and preparing for their winter's work. The hot months, relaxing alike to the mental and physical energies, have caused a cessation of society work for a time. But now that the profession is settling down once more to its regular labors, the demands of the local medical societies upon the recognition and attention of the medical man should not pass unheeded. It is to be regretted that the advantages of such associations for united professional work are not more fully recognized, or if recognized, that such recognition is not more universally translated into practice. That medical societies are the most potent instrumentalities for the advancement of medical science is a fact that cannot be gainsaid by any observant man. A moment's thought will convince any one that the best results that are obtained in this direction are almost invariably wrought out under the stimulating influence of the contact of mind with mind. The practitioner is thereby lifted out of the ruts into which every one is so liable to fall when working by himself alone, and he learns that there are other works of practice and other lines of thought outside of those to which he has become accustomed. The tendency of society work is to broaden a man's views of medical matters, to enlarge his field of vision, and thereby to make his daily practice more satisfactory to himself and more beneficial to his patients.

These are facts which are too potent to admit of argument. Yet there are many men of good standing in the profession who are never seen in the medical society. Why this should be so it is difficult to understand. It is true that the arduous duties of a large practice leave but little leisure for anything else; yet the men who have large practices are the very ones who will derive the most benefit to themselves by occasionally stepping aside from the practical application of their wide experience to compare their views with those of other men and to avoid thereby the tendency to routine which is inseparable from unremitting professional toil. Moreover, the social element which always enters to a greater or lesser extent into all societies is promotive of a friendly professional feeling and a proper *esprit de corps*, which is apt to drop out of sight altogether when every man is working on his own independent schedule.

To the younger portion of the profession the value of the medical society cannot be over-estimated. There he has the opportunity to avail himself of the experience of older and wiser men and to learn lessons in practice which cannot be learned from the generalizations of his text books. Next to actual individual experience there is no school so valuable to the young practitioner as the medical society.

We would therefore urge upon our readers the desirability of the formation and support of organizations of this kind. In thinly settled districts two or more counties might advantageously unite and hold monthly meetings at some accessible point. In the cities and larger towns where societies already exist we would urge a more general membership and a more frequent attendance of meetings. The results of such a course would soon be felt in an elevation of the tone of the profession, a promotion of scientific and accurate medical knowledge, and a better ethical spirit everywhere. Petty jealousies and animosities would disappear, knowledge of medical science would be promoted and the profession would be brought nearer to that plane which we all recognize as alone worthy of a noble and lofty calling.

